# Nurses’ Challenges With Personal Protective Equipment: A Qualitative Content Analysis Based on the COVID‐19 Pandemic

**DOI:** 10.1155/nrp/9965413

**Published:** 2026-02-13

**Authors:** Masoomeh Imanipour, Halimeh Kamali

**Affiliations:** ^1^ Department of Critical Care, Nursing and Midwifery Care Research Center, School of Nursing and Midwifery, Tehran University of Medical Sciences, Tehran, Iran, nosm.ca; ^2^ Health in Disasters and Emergencies Research Center, Institute for Futures Studies in Health, Kerman University of Medical Sciences, Kerman, Iran, kmu.ac.ir

**Keywords:** COVID-19, nurses, personal protective equipment

## Abstract

**Objective:**

In pandemic conditions, paying attention to the facilities and amenities nurses need while caring for patients is important. In this regard, this study aimed to explore the challenges and problems faced by nurses regarding the use of personal protective equipment during the COVID‐19 pandemic.

**Methods:**

This is a qualitative study with conventional content analysis using Granheim and Landman’s approach. In this study, 12 participants were selected by purposive sampling, considering the inclusion criteria. Data were collected through in‐depth semistructured interviews. Ethical considerations were applied to all stages of the study. MAXQDA 20 software was used to manage and organize the data.

**Results:**

About 59% of the participants (7/12) were female. The mean age of the participants was 37 years, and work experience ranged from 7 to 22 years. One main theme includes “Unfavorable management of personal protective equipment,” and seven categories emerged from the data analysis.

**Conclusion:**

Nurses face numerous challenges and problems in epidemics and pandemics, such as the COVID‐19 pandemic. As these people are on the front lines of the fight against the pandemic, they always need various equipment and facilities, including qualified and standard personal protective equipment. Therefore, special attention should be paid to these members of the healthcare team through providing practical approaches to mitigate their challenges about using personal protective equipment.

## 1. Introduction

Emerging diseases often reactivate periodically and spread rapidly through susceptible populations, eventually reappearing and spreading rapidly, and ultimately developing into epidemics or pandemics worldwide [1]. The first decade of the twenty‐first century saw three significant emergencies related to emerging diseases: severe acute respiratory syndrome (SARS) in 2002–2003, avian influenza in 2006, and H1N1 in 2009 [2]. The Middle East respiratory syndrome (MERS) in 2012 and Ebola in 2014–2016 were other epidemics in the twenty‐first century [3, 4]. More recently, the COVID‐19 pandemic has spread worldwide and become a global pandemic. In epidemic situations, nurses are considered the mainstay of the health system response [1] and are on the front lines of the fight, putting their lives at risk [5]. However, they are at increased risk due to the rapid spread and transmission of the disease [6, 7]. Preventative strategies, such as wearing personal protective equipment (PPE), practicing hand hygiene, and maintaining physical distance, have been essential in minimizing the transmission of COVID‐19 and have played a crucial role in safeguarding healthcare workers [8].

Essentially, the main strategy for preventing the transmission of highly infectious diseases, such as COVID‐19, is using PPE. PPE can significantly reduce the risk of contamination and infection [9]. PPE includes masks, hats, gowns, coveralls, gloves, shoes, goggles, face shields, and ear protection [10]. This PPE will only be useful if it can prevent the transmission of contaminated body fluids between healthcare workers, patients, and others [10]. However, these devices must be manufactured to hygiene standards to be impervious to fluids and viruses and technically prevent skin contamination [10]. However, following the outbreak of COVID‐19, problems and challenges related to this equipment were reported in the health systems of countries around the world [7]. Recent studies in this area, focusing on the use of PPE, risk awareness, and experiences of healthcare workers, have been conducted in countries, such as Korea [11], Nigeria [12], Bangladesh [13], and Australia [14]. Based on the researcher’s experience serving as a nurse providing care in the COVID‐19 intensive care unit (ICU), Iran was no exception in this regard.

Nurses are considered important members of the healthcare system, and human society needs their effective services in such critical situations [6]. However, COVID‐19 is an unknown traumatic event for nurses. The difficult and exhausting conditions that prevail in caring for patients with COVID‐19 will present nurses with various challenges. Therefore, it is necessary to pay special attention to this group of health system employees and examine the problems and negative consequences of working during a pandemic of an infectious disease in various dimensions, to provide preventive or supportive‐rehabilitative solutions for them. By paying special attention to the work environment’s conditions, the quality of work improves. Additionally, maintaining the health and hygiene of nurses increases productivity. Among the issues that require attention and consideration are the facilities and amenities they need, such as PPE, and the challenges of using these devices while caring for COVID‐19 patients. Nevertheless, there has yet to be a study that outlines the narrative experiences of the nursing workforce’s challenges associated with PPE in Iran, especially during the initial phases of the COVID‐19 pandemic. For this reason, this descriptive qualitative study was designed to examine the challenges and problems faced by nurses regarding the use of PPE during the COVID‐19 pandemic in Iran.

While general information regarding PPE and nursing challenges can be found in textbooks, the nuanced, lived experiences of nurses confronting these issues in the unprecedented context of a global pandemic require a deeper, qualitative exploration. This study was specifically designed using a qualitative content analysis to delve into the rich, detailed accounts of nurses, uncovering the underlying themes, perceptions, and contextual factors that shape their challenges with PPE. A quantitative approach would likely fail to capture the depth and complexity of these experiences, such as the emotional toll, the specific decision‐making processes in resource‐scarce environments, and the unique barriers encountered by different nursing roles and settings.

The qualitative method allows us to move beyond simply documenting the existence of challenges to understanding the “how” and “why” behind them, as articulated directly by the nurses themselves. This approach is crucial for generating insights that can inform more effective interventions, policy development, and practical guidelines for PPE use in future health crises. The researcher hopes that the findings of this study can shed light on these issues and challenges, and thereby enable managers to devise effective and practical solutions to mitigate these challenges.

## 2. Methods and Materials

### 2.1. Study Design

The qualitative research method emphasizes the dynamic, comprehensive, and individual dimensions of human experiences and helps the researcher examine these dimensions from the perspective of the people who have experienced them [15]. To identify the problems and challenges of nurses in using PPE during the COVID‐19 epidemic in Iran, a descriptive qualitative study was conducted. This qualitative study was conducted with a conventional content analysis approach. In qualitative content analysis, narrative data are analyzed to identify and characterize a prominent theme and patterns within this theme [16], and it is useful when the research question involves understanding the meaning, themes, or patterns in the data, as it provides a structured and repeatable way to identify and classify these elements [17]. This study was also prepared with the Consolidated Criteria for Reporting Qualitative Research (COREQ) Standards for qualitative research reports [18].

### 2.2. Participants and Setting

This study was conducted in COVID‐19 general referral hospitals (as the main center for hospitalized COVID‐19 patients) affiliated with the Tehran University of Medical Sciences, Tehran Province, Iran. The study population consisted of nurses caring for COVID‐19 patients. The purposive sampling method was used in this study. In the purposive sampling method of key individuals, participants are selected based on their specific knowledge and understanding of a phenomenon, to share their knowledge and awareness [19]. Inclusion criteria for participants included the following: nurses caring for confirmed COVID‐19 patients who were required to use PPE when working with these patients and have at least one month of experience caring for COVID‐19 patients in COVID‐19 general referral hospitals. The researchers attempted to sample a variety of individuals with different expertise and experiences to obtain accurate and in‐depth information. A total of 12 participants (7 women and 5 men) were enrolled in the study from November 2020 to January 2021.

### 2.3. Data Collection

The data collection method was an in‐depth semistructured interview conducted individually. Before conducting the interview, eligible nurses were consulted, and the date and location of the interview were mutually agreed upon. All interviews were recorded with the participant’s knowledge and permission. To begin the study, the researcher asked an open‐ended and general question, such as “What difficulties do you face in using personal protective equipment?” or “What problems do you endure when wearing this equipment?” to encourage participants to express their experiences. To conduct the interview, an interview guide was used in advance, and in case of unclear points, subquestions and helper questions were also used. The sequence of questions was not the same for all participants and depended on the interview or the interviewee. However, the researcher limited the participants by answering these questions. As the interview program is a dynamic process, limiting it to a number of questions would certainly enrich the data. Despite the fact that the researcher considered the time and tried to return the discussion to the main track in case the participants deviated from the main topic, he had to establish with the company founders and adjust the questioning process based on their statements. The information was in the context of a request for information. Finally, participants were asked to indicate if they wanted to provide any further explanations regarding the challenges and problems experienced in this context. Other probing and exploratory follow‐up questions such as “Can you explain more?” or “Can you give an example?” and “What do you mean?” were asked based on the data provided by the participants to clarify the concept and deepen the interview process. So, different dimensions of the challenges that nurses face when using PPE were explored. These dimensions included individual dimensions in terms of physical or psychological problems, as well as organizational dimensions.

Sampling continued until data saturation was reached and was carried out with maximum diversity in terms of age, gender, length of care for COVID‐19 patients, and work sector. The data saturation occurred after conducting ten interviews. Data saturation is commonly used in qualitative research methods, such as interviews, focus groups, content analysis, or ethnographic studies [20]. During the data analysis phase, researchers determine whether data saturation has been achieved by assessing if new findings offer unique insights or merely repeat previously gathered information. To confirm data saturation, two additional interviews were conducted. All interviews were conducted by the first author, who had sufficient experience in conducting interviews. Interviews lasted between 40 and 90 min.

### 2.4. Data Analysis

The approach proposed by Granheim and Lundman for qualitative content analysis was used [21]. The researcher transcribed the interviews and studied them several times to gain a complete understanding of them. The entire interviews were considered as the unit of analysis. The unit of analysis refers to the notes that were to be analyzed and coded [21]. Paragraphs, sentences, or words were considered the semantic unit. A semantic unit is a set of words and sentences that are related to each other in terms of content and are summarized and placed together according to their content and implications [21]. Then, the semantic units were conceptualized at the level of abstraction and named by codes according to the meaning hidden in them. The codes were compared with each other in terms of their similarities and differences and were categorized under more abstract categories with specific labels. Finally, by comparing the categories with each other and reflecting on them carefully and deeply, the hidden content within the data was introduced as the theme of the study.

In addition, data analysis was carried out simultaneously and continuously alongside the data collection process. For initial coding, the researcher used participants’ statements and index codes derived from the interviews. Initial codes were extracted from the interviews as meaningful units of participants’ statements. These codes were reviewed several times and categorized based on their similarity and relevance. Subsequently, similar codes were merged, and the categories were further refined, leading to second‐level coding. In the next stage, the categories were compared with each other and categories that had similar characteristics were combined to create broader categories. The codes obtained from data analysis were continuously reviewed and revised until the final stages of writing the study. The initial extracted codes were reduced during ongoing data analysis and comparison, leading to the finalization of the main concept and categories related to nurses’ challenges related to the use of PPE in the COVID‐19 pandemic. MAXQDA 20 software was used to code and extract categories and themes.

### 2.5. Trustworthiness

To ensure data validity and reliability or quality control, Lincoln and Guba’s (1985) four criteria are used, which are credibility, dependability, confirmability, and transferability [22].

#### 2.5.1. Credibility

Credibility refers to activities that increase the likelihood of producing valid findings, and one of the best methods for credibility is long‐term engagement with the subject [23]. In order to increase the credibility of the research, the researcher tried to make the data and findings deeper and more authentic by spending enough time collecting and analyzing data and selecting experts with different specializations and experiences. Also, in this study, various integration methods were used, including data integration (spatial integration and integration of individuals) and integration of methods by using multiple methods for collecting data, including interviews, recording reminders, and field notes; along with sampling with maximum diversity in terms of age, gender, occupation, education, and work experience, the credibility of the research results was increased, in addition to describing the context under study, providing necessary explanations about the participants, and using their direct quotes.

#### 2.5.2. Dependability

Peer review is a method to increase the dependability of the data. In this study, the first three interviews and then randomly selected interviews, along with the results of the data analysis, were sent to a number of nursing professors and colleagues to express their opinions during the research process. After reviewing the first three interviews, the interview guide questions were changed and the coding method was finalized based on the opinions of the peers.

#### 2.5.3. Confirmability

The audit method was used for confirmability. To increase the verifiability of the data, the researcher fully described all the stages of the research (data collection, analysis, and formation of variables and concepts) so that, if necessary, another person could follow this process and understand the why and how.

#### 2.5.4. Transferability

Transferability or transferability means the likelihood that the findings will have a similar meaning for others in a similar situation. The nature of qualitative studies affects the generalizability of research results. In qualitative research, more attention is paid to the extent to which the information obtained is representative of all available information, rather than to the representativeness of the samples. To ensure data transferability, the stages of the study and the activities carried out in the research process were written in detail with clear and purposeful descriptions (detailed description of the participants, sampling method, time and place of data collection, and method of data collection and analysis), so that other people in different places can do it. In addition, the purposeful sampling with maximum diversity used in the qualitative part of this study helps to ensure transferability.

Finally, for reflective thinking or researcher’s reflexivity, the researcher has a nursing background and a recent master’s degree in nursing with a major in adult critical care and several years of clinical nursing experience and training as an instructor in teaching internal and surgical nursing units and critical care, in health centers and hospitals. The above experiences provided the researcher with the opportunity to conduct better and more accurate research in this area and to gain a deeper understanding of the phenomenon under study. During the research, the researcher, by continuously performing the bracketing process, wrote down on a page the tendencies and thoughts that influenced the flow of the interviews and tried to prevent the influence of his own views and attitudes during the research process as much as possible.

### 2.6. Ethical Considerations

This study was approved by the Ethics Committee of Tehran University of Medical Sciences (IR.TUMS.FNM.REC.1399.145), and permission was obtained to conduct the study. This study followed the ethical guidelines set forth in the Declaration of Helsinki. The researchers introduced themselves to the participants and clearly explained the objectives of the study. Written informed consent was obtained for participation in the study and audio recording. The location and time of the interview were selected based on the participants’ preferences. Participants were assured of the confidentiality of their data throughout the research process and were allowed to withdraw from the study at any time. In addition, participants were encouraged to contact the researchers if they had any questions or concerns.

## 3. Results

### 3.1. Sociodemographic Characteristics

In this study, the average age of the participants was 37 years. About 59% of participants were female. The work experience of the nurses in this study ranged from 7 to 22 years, and in terms of education, 58% had a bachelor’s degree in nursing. Most study participants were shift managers or ICU nurses (Table 1).

**Table 1:** Demographic characteristics of participants.

Demographic characteristics	Number (percentage)
Gender	
Male	5 (41)
Female	7 (59)
Age	
25–34	6 (50)
35–44	4 (34)
45 and above	2 (16)
level of education	
Bachelor’s degree	7 (58)
Master’s degree	2 (17)
Ph. D in nursing	3 (25)
Position	
Clinical supervisor	1 (8)
Nurse manager	1 (8)
Shift manager	3 (25)
Registered nurse	7 (58)
Work experience (years)	
5–10	5 (42)
11–15	4 (33)
16–20	2 (17)
21 and above	1 (8)

### 3.2. Qualitative Result

Based on the analysis of the interviews, the main concept “Unfavorable management of personal protective equipment” was identified. The extracted evidence was collected in 7 main categories including Poor access to equipment, Providing nonstandard equipment, Improper management of contaminated equipment, Undesirable use, physical intolerance, psychological intolerance, and Adverse consequences in caring, which are explained below with quotes from the participants (Table 2).

**Table 2:** Main concept, categories, and evidence extracted from qualitative content analysis.

Main concept: unfavorable management of personal protective equipment	
Categories	Codes

Poor access to equipment	Delayed provision Equipment scarcity Provision of equipment through need declaration Equipment rationing Sign and confirm receipt of equipment Equipment provision through donor support Unfair distribution

Providing nonstandard equipment	Poor quality of equipment Incompatibility of equipment size with body physique Provision of quality equipment at personal expense

Improper management of contaminated equipment	Lack of protocol for contaminated equipment

Undesirable use	Lack of monitoring of usage Lack of proper usage protocol Lack of knowledge on correct usage Lack of training for correct usage Intentionally not using equipment

Physical intolerance	Physical consequences Disruption in fulfilling essential needs

Psychological intolerance	Psychological consequences Disruption in fulfilling spiritual needs

Adverse consequences in caring	Disruption in providing care Disruption in communication

### 3.3. Category 1: Poor Access to Equipment

Based on the statements of the participants in this study, it was determined that the supply and distribution of PPE was unsatisfactory, and the performance of the relevant managers was reported to be poor. The participants stated that in the early stages of the COVID‐19 outbreak, PPE was not provided on time, and some also stated that the managers provided this equipment after the announcement of the dire need and the insistence of the nurses at the highest risk.

As Participant Number 12 stated: “Well, at the beginning of the pandemic, there was a lot of opposition to providing this equipment. when the staff in the ICU department put pressure on the nursing manager and both the supervisor and the head nurse, they decided to provide us some PPE” (P12).

Most participants in this study stated that they faced the challenge of a shortage of PPE in the early days of the COVID‐19 pandemic. As participants stated: “The problem we had at first was the lack of personal protective equipment, it was very little” (P3). “There was only one simple mask and a simple gown in the unit” (P9). “We admitted the first COVID‐19 patient on February 18, and we didn’t even have personal protective equipment, only two or three.” (P11). “It was very difficult for us at first. In the first few weeks, we were severely lacking in equipment, and sometimes it even caused us various problems. A few N95 masks were distributed, but it was still not enough” (P8).

A number of participants stated that the lack of equipment led to the distribution of equipment in a rationing manner. The rationing method was also implemented based on the number of staff, shift patterns, and the number of patients under care. Participants stated: “When they buy the equipment in total, they give it to the nursing manager. he would divide it according to the number of staff in each shift. for example, in the morning shift, one simple mask, and one N95 mask for each nurse” (P1). “They give us three or four pairs of gloves. The night shift is long and the work we do is more. They give eight pairs for two patients. That means four pairs for each patient. We often face a shortage of gloves” (P7).

Some participants even stated that at the beginning of the pandemic, PPE distributed to staff had to be signed and confirmed by them, and then returned. As Participant Number Two explained: “We had to sign when we received personal protective equipment, for example. But not masks. It wasn’t like that and we changed them every shift, but the clothes were returned” (P2).

Participants in this study stated that most of the time, PPE was provided by donors and distributed by hospital administrators to staff and nurses. Participant 10 stated: “A lot of this equipment was provided by donors. That is, they would give the money to the hospital and the hospital would provide it for us” (P10).

Some participants believed that PPE was not provided for all job categories or was not distributed fairly among staff. All of them were in contact with the disease in different ways. Participant number 4 expressed this: “Our auxiliary forces who came and worked in the COVID‐19 ward did not give them N95 masks at first. They only gave them to those of us who were nurses” (P4).

### 3.4. Category 2: Providing Nonstandard Equipment

In the early days of the COVID‐19 pandemic, most of the PPE that was provided was of poor quality and durability. As participants stated: “They were of poor quality, very thin and extremely hot because the fabrics used were of poor quality but waterproof. It had elasticated cuffs. It had a full‐face helmet but it was completely unusable and we were sweating within the first half hour and it was extremely hot” (P3).

Participants also reported that during the COVID‐19 pandemic, PPE was provided for personnel, but unfortunately, it was not of the appropriate size and standard. For example, participants stated: “In terms of size and shape, they were all the same size, except for the XL inside. We had a very small force, it didn’t have clothes for it.” (P1). “There was no standard size. They bought everything in bulk, they mostly made something so that it would actually fit the men’s body, and sometimes they bought a medium size and the men would wear it. God knows these pants would come down to below the knee, and they would laugh at themselves and wear them over those surgical pants so that their feet wouldn’t be visible. I don’t know what the problem is, but the clothes we have now are not quality clothes” (P7).

Participants stated that due to poor procurement processes for PPE or the distribution of poor quality equipment, staff sometimes procured quality equipment themselves to attend work shifts. Participant 12 stated: “The hospital gave us shields that were poor quality shields. That is very short shields that did not provide much coverage. It was clear that they were of very poor quality. I procured shields myself from outside. A standard shield with a long edge that I could work with safely” (P12).

### 3.5. Category 3: Improper Management of Contaminated Equipment

Participants stated that there was no written protocol regarding the process of collecting and disposing of contaminated PPE, and that standard environmental conditions for disposing of contaminated PPE were not observed, as Participant No. 4 stated: “The clothes they gave us when we first arrived, they said, ‘You go inside the locker room’. Unfortunately, our locker room is shared in the ICU… That is, the ladies and the gentlemen… That means we have to wait for them to come out and then we go inside… They had set a time limit for the nurses to put on their clothes there, and the service would take these clothes to the lingerie room for each shift, and then you had to go and get clean lingerie and come back to the ward and wear them. Then they said, ‘No, the locker room is getting contaminated…’ When everyone finishes their shift, they take their clothes down, hand them in, and then again…” (P4).

### 3.6. Category 4: Undesirable Use

Participants in this study stated that managers do not monitor how nurses use PPE. As Participant 5 commented: “There is no monitoring. When we enter the ward, we receive our belongings before we take off our clothes, go to the clean changing room, change our clothes, no more monitoring after that” (P5). Participant 9 also stated: “We give everyone a share of the mask, and they know whether to change it or not, so there is no more monitoring from that point on” (P9). Some participants also reported that there is no written protocol regarding the basic use. As Participant 3 stated: “The hospital was not prepared for such a crisis and we did not have PPE. I did not wear a mask until then, except for suctioning, and I only used a mask when I was suctioning, and then the coronavirus came and spread all of a sudden” (P3).

The participants stated that the disease was unknown and they did not have the necessary knowledge to deal with it, so they were also misled and ignorant about how to use PPE. Participant number 1 stated: “Since we still did not know what it was, how it was transmitted, how long it lasted in the environment… and many other things… At first, everything was misleading… People did not know exactly what to do… Even in the community, if you remember, they said that no one needed to wear a mask, but then they said that only the medical staff in the hospital should wear masks… All this made people not know where to wear a mask, where to wear a shield, and where to wear gloves” (P1).

Also, based on the analysis of the interviews of the participants of this study, it was clear that there was no sufficient and documented training provided on how to use this equipment. For example, participants stated: “I didn’t get any training, at least not in my department. No one taught us. they learned everything …they learned, either from each other or from searches or clips they found from different places” (P12). “No, they didn’t teach us how to put on things and how to take them off. Everyone learned it themselves or from each other. No… We didn’t get that much training” (P3). “There was no training… even how to put on a mask… I remember in the beginning, some people said that the mask should have a white part on the outside or a blue part on the outside… In short, everyone came and gave a thesis… Then you thought to yourself, who is right?” (P11).

Some participants also stated that they deliberately did not use personal equipment, as participant number 12 stated: “These clothes of ours have a cap and a strap, and they are beautiful… but the men do not use them. I did not use them because I have hair loss and my philosophy is that it is due to sweating, so I did not use them and I still do not use them” (P12).

### 3.7. Category 5: Physical Intolerance

Most nurses complained about the difficulty of working with PPE and the physical intolerance of these devices. The use of such devices caused physical problems and consequences. Nurses mentioned skin problems, headaches, nausea, dehydration, etc., due to the use of PPE while caring for patients. Participants stated: “I had a lot of shortness of breath, so my colleague did several HRCTs because of this, but then it turned out that the shortness of breath was due to using the mask” (P9). “I remember when I put it on, I put it on over my coat first. I started sweating heavily and went to take off my coat. I lost a lot of weight because I was sweating, I lost about 6 pounds during the coronavirus. At first, I used to sweat and get nervous. To be honest, I get nervous easily when it’s hot” (P6). “Usually, when we are on shift, a few hours after we get back from the shift, both the volume of our urine has decreased a lot and it has become a bit more yellow because we use less fluids and the first day is still harder because we use more fluids at home. The second day after night shift and the third day after night shift will be a bit better” (P3). “We had two types of gloves. Their name is latex and the other one I forgot the name of. These latex gloves were good. I’ll ask them their name again and I’ll tell you. They were very allergic and whenever we put them on our hands, we would get itchy. It was very annoying during the shift. They had powder inside them. That powder inside them caused allergies. Maybe 90% of our colleagues would get itchy hands” (P4).

Several participants reported that the use of PPE interfered with meeting their physical needs. They stated that to meet physical needs, such as drinking water or using the bathroom, they had to completely remove and then put on PPE again, which was a source of discomfort for them. Therefore, participants stated: “They often drink less fluids so that they don’t have to go to the washstand, so they have to take off these covers so that they can go to the washstand, which is a bit difficult for them” (P5). “they sometimes prefer to drink less fluids or not drink as much juice so that they don’t have to take off these clothes to go to the washstand” (P10).

### 3.8. Category 6: Psychological Intolerance

Other problems reported in this study were psychological problems and consequences for nurses. In this regard, participants stated: “At first, I was a little embarrassed when I wore these clothes. It was like, ‘The clothes are a little big for me, and they don’t look good’” (P7). “I was like, ‘How am I going to wear these clothes? Put on a mask, put on a hat, put on glasses. At first, I feel embarrassed about how I’m going to work with these’” (P10).

There are also complaints about the participants’ spiritual needs being met. Participants commented: “I didn’t fast for the first couple of days, and now I was afraid. I didn’t fast for maybe two or three days. With the summer heat, I had a hard time fasting” (P2). Participants also stated that they had to completely remove their protective equipment to perform ablution and then do it again. Participant number 11 also did this: “Yes, if they wanted to go to the washstand and perform ablution because they had gone through the same steps, sometimes they would delay and the prayer would be delayed and postponed until the end of their shift.” (P11).

### 3.9. Category 7: Adverse Consequences in Caring

Participants stated that the use of PPE interfered with the provision of patient care, often compromising the quality of care, slowing down patient care, and sometimes causing nurses to stop using PPE. Participants reported: “Because they were so heavy, and these clothes were holding your hands and feet. It slowed down your speed” (P5). “Walking in those boots was very heavy and difficult for them. It’s almost like no one else in the ICU uses boots at all” (P3).

Some participants also stated that the use of PPE interfered with verbal, nonverbal, and therapeutic communication with the patient. As participants reported: “One of the principles I taught in the hospital was that when you open the door to the patient’s room or go to the patient’s bedside, you should always go with a smile so that the patient can see your positive energy and your face and that way they should be happy. However, with these masks, they don’t see a smile from the nurse because they are all covered” (P6). “The patient can’t even make that eye contact because of the shield and glasses, etc., and nonverbal communication has decreased” (P12). “That look you sometimes make to patients, winking at them, laughing, they don’t see your smile, they don’t even see your face, they don’t see eye contact. You can’t even do the therapeutic touch with the gloves on your hands all the time, because of the nonverbal communication that is missing” (P11). “At first, it was a bit difficult for the patients. They didn’t know us. They didn’t know if a gentleman was going to their room or a lady. I mean, one of the challenges that I was talking about with them was the ethical challenges. When we went to the patient’s room, until you spoke, they didn’t understand whether it was a lady or a gentleman. Because we had shields, masks, glasses, and hats, they didn’t understand. Then we decided to write all our names on our clothes with a marker” (P3). Finally, Participant Number 8 stated: “ for almost conscious patients, the personal protective equipment is both scary and they cannot distinguish their nurses from others. On the other hand, we all look the same… and I even think it can make the patient anxious and increase the risk of delirium” (P8).

## 4. Discussion

Nurses face a higher likelihood of acquiring infectious diseases, including COVID‐19, as a result of their direct interactions with highly infectious patients, as well as their potential exposure to undiagnosed or asymptomatic infections. This issue is exacerbated by the widespread lack of access to PPE globally [24]. Nurses have not been examined as a group regarding asymptomatic carriers and individuals who are at risk due to the asymptomatic spread of the coronavirus during the recent COVID‐19 pandemic [25]. Consequently, this study sought to elucidate the difficulties nurses encounter in utilizing PPE throughout the COVID‐19 pandemic and pinpointed a core concept of the “unfavorable situation of nurses concerning personal protective equipment” which was subsequently examined in depth.

Personal safety is a primary concern for nurses when dealing with a contagious and threatening illness, such as COVID‐19. To effectively carry out their care responsibilities, nurses must ensure that they have access to the necessary facilities and equipment to safeguard their health from the virus and maintain the quality of their care. However, many nurses in this study reported significant shortages and inadequate access to equipment that meets optimal quality and standards early in the pandemic. The unavailability of PPE is a challenge that healthcare facilities face during pandemics. This concern was also highlighted in the research conducted by Wang et al. An outbreak caused by a novel pathogen can frequently overwhelm the healthcare system, leading to a depletion of resources and medications [24]. For instance, in 2009, the United States experienced an outbreak of H1N1 influenza. Numerous hospitals encountered a lack of PPE [24].

During the COVID‐19 pandemic, Wang et al. indicated that the shortage of equipment was attributed to the overlap of the Chinese New Year with the pandemic’s outbreak, as most manufacturers and distributors were on holiday at that time, which intensified the lack of medical protective gear in combating the epidemic [24]. Hospitals throughout China, particularly in Wuhan, were experiencing an extremely critical scenario and encountered a significant lack of medical supplies, notably personal protective gear, such as medical suits and N95 masks. Hospitals urgently appealed for community assistance [24]. Iran encountered a similar scenario. Throughout the nation, donors rallied to support hospitals and medical facilities, attempting to alleviate the impact of these challenges by supplying PPE. The researcher observed these efforts firsthand while working as a registered nurse in an ICU for COVID‐19 patients. From the researcher’s perspective, during the initial phases of the COVID‐19 pandemic, the urgency of the illness and the acute lack of personal protective gear sometimes led to donors providing equipment to hospitals, which often turned out to be of substandard quality.

Throughout the COVID‐19 pandemic, numerous healthcare systems faced challenges in supplying sufficient PPE owing to budgetary or time limitations [26]. The lack of PPE has resulted in the adoption of strategies, such as reducing, reusing, or opting for substandard or unapproved products in an effort to cope with the shortfall of these essential items [7]. Although having sufficient equipment is essential in combating COVID‐19, significant actions need to be taken by healthcare administrators and government entities to guarantee that medical professionals, including nurses, have access to necessary supplies [26]. Moreover, there have been instances of PPE being taken from healthcare facilities, improperly used, or stockpiled, which could hinder the availability of these supplies for those most at risk [27] and even force them to work without it [26]. The examination of the evidence in this research showed that during the COVID‐19 pandemic, a lack of equipment forced nurses to reuse PPE throughout their shifts, and the distribution of this equipment was limited. Even at the onset of the COVID‐19 pandemic, PPE was frequently provided on a delivery basis, requiring nurses to sign for it upon arrival. Another issue is the unequal access to PPE for high‐risk members of the healthcare team [27]. In this study, nurses expressed that PPE was not available in adequate amounts for all roles, and even when it was supplied, it often did not meet the necessary quality standards. Nevertheless, safeguarding the healthcare workforce is crucial to ensure ongoing patient care, not just for doctors and nurses or paramedics, but also for other support personnel, given the threat of infection to additional healthcare team members.

The actions of managers and officials throughout this pandemic demonstrate a lack of attention to their duty to properly stock medical supplies necessary for epidemic prevention and control, as well as to establish protocols and guidelines. Additionally, appropriate training and documented procedures on how to utilize this equipment were not provided by the relevant authorities. Moreover, some research has suggested that healthcare workers’ compliance with the use of PPE is unsatisfactory, even though they are aware of the risks associated with the transmission of pathogens [28]. A study conducted by Brown, Monroe, and Rogers (2019) identified several obstacles to optimal adherence among healthcare workers, including a low perception of risk, time constraints, fatigue, insufficient staff training, and a lack of personnel [28]. Essential for nurses during epidemics is their comprehension of the role, kinds, and correct application of PPE. Also, based on the study by Nasrabadi et al. (2021), conducted in Iran to investigate the reasons for the nonuse of PPE among healthcare workers during the COVID‐19 pandemic, it was found that low supervision and lack of information are key factors contributing to the nonuse of PPE [29]. This is why, a rational grasp of how to use personal protective gear allows nurses to select the most suitable approach when caring for patients, as the efficient utilization of these tools safeguards both nurses and patients from the transmission of infectious pathogens [28].

Conversely, during critical events and outbreaks, like the rapid spread of acute respiratory syndromes, such as coronavirus and SARS, nurses experience significant mental and physical strain due to inadequate time for rest and recovery, alongside constant exposure to stressors [5]. Images showing the faces of medical staff, marked by prolonged mask usage, have been utilized to highlight the challenging circumstances they face while treating these patients. These circumstances are further compounded by discomfort, pressure, overheating, and fluid depletion resulting from the use of respiratory protection gear [7]. Research in this field encompasses Kang’s study from 2018 and Fu’s from 2006. As noted by Kang et al., issues concerning PPE among nurses involve unsuitable sizes of equipment, feelings of anxiety, uncertainty regarding nonstandard protocols, concerns about the quality and efficacy of the equipment, and difficulties in using it [30]. Foo et al. also examined skin issues related to the use of this equipment, and the results of their study indicated that the utilization of items, such as masks and gloves by nurses, leads to common skin conditions (acne, itching, rashes, and dryness) [31]. Moreover, the extended use of PPE without a break can heighten anxiety and confusion, aligning with the results of this study. Maghsoodi et al. reported in their study that using PPE for a long time caused side effects, such as fatigue, sweating, mask marks on the face, back pain, varicose veins, headache, and overall physical discomfort [32]. Participants in this research also reported experiencing both physical and psychological issues and effects. This is consistent with the study by Manookian et al., which is stated in this study while this gear offers significant protection for healthcare workers in combating infections, such as COVID‐19; in practical settings, the use of PPE can be inconvenient and unwieldy for healthcare workers [33]. Such discomforts, if not adequately managed, may lead to reduced compliance with PPE usage, particularly during extended periods of wear [33]. As a result, these elements can jeopardize the well‐being and safety of healthcare workers, hinder their physical capabilities, and complicate their tasks, which may result in adverse effects on patients and potentially jeopardize their safety [34].

In summary, enhancing the national public health emergency response framework is crucial for the prevention and management of emerging infectious diseases, and medical equipment for emergencies, particularly personal protective gear, plays a crucial role in public health emergency response [26]. In the coming years, Iran and various other nations ought to closely monitor the program for stockpiling medical equipment as part of enhancing the public health emergency response system, drawing lessons from the COVID‐19 pandemic and from the management of other outbreaks. Initiatives for the supervision, storage, deployment, distribution, rapid production, and emergency requisition of critical supplies should be advanced through changes in legislation. Additionally, informed by the results of this study and by concentrating on the various dimensions of the challenges in this area, they should strategize to tackle issues related to the potential for future pandemics. Establishing regional and national strategic stockpiles with clear protocols for activation can ensure rapid access to essential PPE. Public–private partnerships can play a pivotal role in scaling up production capacity and ensuring supply chain resilience, particularly for critical components, such as N95 respirators and medical‐grade gloves. Countries should also prioritize workforce training and simulation exercises to ensure healthcare providers are prepared to deploy and manage emergency supplies effectively. Finally, incorporating feedback from frontline workers and incorporating community‐based surveillance can improve responsiveness and adaptability in future health emergencies.

### 4.1. Limitations

One notable limitation of qualitative research is its intrinsic subjectivity and the possible effect of researchers’ biases on the findings. In this investigation, the researchers sought to recognize their pre‐existing beliefs, attitudes, and personal experiences, documenting these elements to lessen their influence on data analysis and interpretation. Furthermore, some potential participants expressed reluctance to engage in the study. To counter this challenge, the interviewer aimed to foster cooperation by elucidating the research objectives and assuring the confidentiality and privacy of the individuals being interviewed. Another constraint was the likelihood that participants might be unavailable for the interview. To address this issue, the aims and importance of the study were communicated to the individuals, and an interview schedule was organized according to their preferences.

## 5. Conclusion

Epidemics of new infectious diseases are spreading rapidly globally, and these epidemics certainly have a significant impact on economic development and human health. However, nurses face numerous challenges and problems in epidemics and pandemics, such as the COVID‐19 pandemic. As these people are on the front lines of the fight against the pandemic, they always need various equipment and facilities, including qualified and standard PPE. Therefore, special attention should be paid to these members of the healthcare team through providing practical approaches to mitigate their challenges about using PPE.

## Author Contributions

The study’s concept and design were created by Masoomeh Imanipour and Halimeh Kamali. Data analysis and manuscript writing were handled by Halimeh Kamali. The research was overseen and critical feedback on the manuscript was provided by Masoomeh Imanipour. The final manuscript was read and reviewed by all of the authors.

## Funding

This study received financial support from the Nursing and Midwifery Care Research Center of Tehran University of Medical Sciences (GRANT NUMBER: 99‐2‐160‐49787).

## Disclosure

The final document was read and approved by the authors.

## Ethics Statement

This study was approved by the Ethics Committee of Tehran University of Medical Sciences (IR.TUMS.FNM.REC.1399.145).

## Conflicts of Interest

The authors declare no conflicts of interest.

## Data Availability

The data used to support the study’s findings are available from the corresponding author upon request.

## References

[1] Devnani M. , Factors Associated With the Willingness of Health Care Personnel to Work During an Influenza Public Health Emergency: An Integrative Review, Prehospital and Disaster Medicine. (2012) 27, no. 6, 551–566, 10.1017/S1049023X12001331, 2-s2.0-84870431586. 23031432

[2] Khabbaz R. , Bell B. P. , Schuchat A. et al., Emerging and Reemerging Infectious Disease Threats, Mandell, Douglas, and Bennett’s Principles and Practice of Infectious Diseases. (2014) .

[3] Wang C. , Horby P. W. , Hayden F. G. , and Gao G. F. , A Novel Coronavirus Outbreak of Global Health Concern, Lancet. (2020) 395, no. 10223, 470–473, 10.1016/S0140-6736(20)30185-9. 31986257 PMC7135038

[4] Kim J. S. and Choi J. S. , Factors Predicting Clinical Nurses’ Willingness to Care for Ebola Virus Disease‐Infected Patients: A Cross‐Sectional, Descriptive Survey, Nursing and Health Sciences. (2016) 18, no. 3, 299–305, 10.1111/nhs.12269, 2-s2.0-85028249307. 26661457

[5] Kim J. S. and Choi J. S. , Factors Influencing Emergency Nurses’ Burnout During an Outbreak of Middle East Respiratory Syndrome Coronavirus in Korea, Asian Nursing Research. (2016) 10, no. 4, 295–299, 10.1016/j.anr.2016.10.002, 2-s2.0-85008433307. 28057317 PMC7104920

[6] Namdari S. , Nasiri A. , Nakhaee S. , and Taheri F. , Exploring the Effects of Nurses’ Family-Work Conflict on Patient Care Quality: A Qualitative Study, Modern Care Journal. (2019) 16, no. 1, 10.5812/modernc.86130.

[7] Tabah A. , Ramanan M. , Laupland K. B. et al., Personal Protective Equipment and Intensive Care Unit Healthcare Worker Safety in the Covid-19 Era (Ppe-Safe): An International Survey, Journal of Critical Care. (2020) 59, 70–75, 10.1016/j.jcrc.2020.06.005. 32570052 PMC7293450

[8] Cohen J. and van der Meulen Rodgers Y. , Contributing Factors to Personal Protective Equipment Shortages During the Covid-19 Pandemic, Preventive Medicine. (2020) 141, 10.1016/j.ypmed.2020.106263. PMC7531934 33017601

[9] Alhazzani W. , Møller M. H. , Arabi Y. M. et al., Surviving Sepsis Campaign: Guidelines on the Management of Critically Ill Adults With Coronavirus Disease 2019 (Covid-19), Intensive Care Medicine. (2020) 46, no. 5, 854–887, 10.1007/s00134-020-06022-5. 32222812 PMC7101866

[10] Verbeek J. H. , Rajamaki B. , Ijaz S. et al., Personal Protective Equipment for Preventing Highly Infectious Diseases Due to Exposure to Contaminated Body Fluids in Healthcare Staff, Cochrane Database of Systematic Reviews. (2020) 4, no. 4, 10.1002/14651858.CD011621.pub4. PMC7158881 32293717

[11] Min H. S. , Moon S. , Jang Y. , Cho I. , Jeon J. , and Sung H. K. , The Use of Personal Protective Equipment Among Frontline Nurses in a Nationally Designated Covid-19 Hospital During the Pandemic, Infection and Chemotherapy. (2021) 53, no. 4, 10.3947/ic.2021.0094. PMC8731245 34951529

[12] Ezike O. C. , Odikpo L. C. , Onyia E. N. et al., Risk Perception, Risk Involvement/Exposure and Compliance to Preventive Measures to Covid-19 Among Nurses in a Tertiary Hospital in Asaba, Nigeria, International Journal of Africa Nursing Sciences. (2022) 16, 10.1016/j.ijans.2021.100385. PMC8650764 34900584

[13] Tune S. N. B. K. , Islam B. Z. , Islam M. R. , Tasnim Z. , and Ahmed S. M. , Exploring the Knowledge, Attitudes, Practices and Lived Experiences of Frontline Health Workers in the Times of Covid-19: A Qualitative Study From Bangladesh, BMJ Open. (2022) 12, no. 1, 10.1136/bmjopen-2021-051893. PMC8753096 35017240

[14] Sundararajan K. , Bi P. , Milazzo A. , Poole A. , Reddi B. , and Mahmood M. A. , Preparedness and Response to Covid-19 in a Quaternary Intensive Care Unit in Australia: Perspectives and Insights From Frontline Critical Care Clinicians, BMJ Open. (2022) 12, no. 2, 10.1136/bmjopen-2021-051982. PMC8819546 35121600

[15] Usman A. C. , Al-Hendawi M. , and Bulut S. , Approaches to Qualitative Research: A Narrative Literature Review, Advances in Medicine, Psychology, and Public Health. (2025) 2, no. 2, 81–95, 10.5281/zenodo.12804998.

[16] Lindgren B.-M. , Lundman B. , and Graneheim U. H. , Abstraction and Interpretation During the Qualitative Content Analysis Process, International Journal of Nursing Studies. (2020) 108, 10.1016/j.ijnurstu.2020.103632. 32505813

[17] Kleinheksel A. , Rockich-Winston N. , Tawfik H. , and Wyatt T. R. , Demystifying Content Analysis, American Journal of Pharmaceutical Education. (2020) 84, no. 1, 10.5688/ajpe7113. PMC7055418 32292185

[18] Dossett L. A. , Kaji A. H. , and Cochran A. , Srqr and Coreq Reporting Guidelines for Qualitative Studies, Jama Surgery. (2021) 156, no. 9, 875–876, 10.1001/jamasurg.2021.0525. 33825809

[19] Aveling E.-L. , Gillespie A. , and Cornish F. , A Qualitative Method for Analysing Multivoicedness, Qualitative Research. (2015) 15, no. 6, 670–687, 10.1177/1468794114557991, 2-s2.0-84946919120. 26664292 PMC4641508

[20] Hennink M. M. , Kaiser B. N. , and Marconi V. C. , Code Saturation Versus Meaning Saturation: How Many Interviews Are Enough?, Qualitative Health Research. (2017) 27, no. 4, 591–608, 10.1177/1049732316665344, 2-s2.0-85013220243. 27670770 PMC9359070

[21] Savin-Baden M. and Major C. H. , Qualitative Research: The Essential Guide to Theory and Practice, 2023, Routledge.

[22] Pandey S. C. and Patnaik S. , Establishing Reliability and Validity in Qualitative Inquiry: A Critical Examination, Jharkhand Journal of Development and Management Studies. (2014) 12, no. 1, 5743–5753.

[23] Gale N. K. , Heath G. , Cameron E. , Rashid S. , and Redwood S. , Using the Framework Method for the Analysis of Qualitative Data in Multi-Disciplinary Health Research, BMC Medical Research Methodology. (2013) 13, no. 1, http://www.biomedcentral.com/1471-2288/13/117, 10.1186/1471-2288-13-117, 2-s2.0-84884171494. PMC3848812 24047204

[24] Wang X. , Zhang X. , and He J. , Challenges to the System of Reserve Medical Supplies for Public Health Emergencies: Reflections on the Outbreak of the Severe Acute Respiratory Syndrome Coronavirus 2 (sars-cov-2) Epidemic in China, Bioscience Trends. (2020) 14, no. 1, 3–8, 10.5582/bst.2020.01043. 32062645

[25] Kursumovic E. , Lennane S. , and Cook T. M. , Deaths in Healthcare Workers Due to Covid‐19: The Need for Robust Data and Analysis, Anaesthesia. (2020) 75, no. 8, 989–992, 10.1111/anae.15116. 32397005 PMC7272944

[26] Ahmed J. , Malik F. , Arif T. B. et al., Availability of Personal Protective Equipment (Ppe) Among Us and Pakistani Doctors in Covid-19 Pandemic, Cureus. (2020) 12, no. 6, 10.7759/cureus.8550. PMC7357309 32670687

[27] Ranney M. L. , Griffeth V. , and Jha A. K. , Critical Supply Shortages—The Need for Ventilators and Personal Protective Equipment During the Covid-19 Pandemic, New England Journal of Medicine. (2020) 382, no. 18, 10.1056/NEJMp2006141. 32212516

[28] Zellmer C. , Van Hoof S. , and Safdar N. , Variation in Health Care Worker Removal of Personal Protective Equipment, American Journal of Infection Control. (2015) 43, no. 7, 750–751, 10.1016/j.ajic.2015.02.005, 2-s2.0-84937400625. 26138659 PMC7132814

[29] Nasrabadi A. N. , Shali M. , Ghorbani A. , Matourypour P. , and Khalilabad T. H. , Challenges With Healthcare Workers’ Protection During the Covid-19 Pandemic in Iran, British Journal of Oral and Maxillofacial Surgery. (2021) 59, no. 3, e114–e117, 10.1016/j.bjoms.2020.10.007. 33579542 PMC7584918

[30] Kang J. , Kim E. J. , Choi J. H. et al., Difficulties in Using Personal Protective Equipment: Training Experiences With the 2015 Outbreak of Middle East Respiratory Syndrome in Korea, American Journal of Infection Control. (2018) 46, no. 2, 235–237, 10.1016/j.ajic.2017.08.041, 2-s2.0-85031508498. 29050907 PMC7115260

[31] Foo C. C. I. , Goon A. T. J. , Leow Y. H. , and Goh C. L. , Adverse Skin Reactions to Personal Protective Equipment Against Severe Acute Respiratory Syndrome–A Descriptive Study in Singapore, Contact Dermatitis. (2006) 55, no. 5, 291–294, 10.1111/j.1600-0536.2006.00953.x, 2-s2.0-33749548018. 17026695 PMC7162267

[32] Maghsoodi E. , Vanaki Z. , and Mohammadi E. , Nurses’ Perception of Work and Life Under Covid-19 Pandemic Conditions: A Qualitative Study, Frontiers in Public Health. (2023) 11, 10.3389/fpubh.2023.1292664. PMC10758251 38164452

[33] Manookian A. , Dehghan Nayeri N. , and Shahmari M. , Physical Problems of Prolonged Use of Personal Protective Equipment During the Covid‐19 Pandemic: A Scoping Review. Nursing Forum, 2022, 57, no. 5, 874–884, 10.1111/nuf.12735. PMC9347983 35575417

[34] Davey S. L. , Lee B. J. , Robbins T. , Randeva H. , and Thake C. D. , Heat Stress and Ppe During Covid-19: Impact on Healthcare Workers’ Performance, Safety and Well-Being in Nhs Settings, Journal of Hospital Infection. (2021) 108, 185–188, 10.1016/j.jhin.2020.11.027. 33301841 PMC7720696

